# Supplementary material for: Comparing strategies for the mitigation of SARS-CoV-2 airborne infection risk in tiered auditorium venues
Source: Commun Eng. 2024 Nov 9;3:161. doi: 10.1038/s44172-024-00297-y (PMC11550442; doi:10.1038/s44172-024-00297-y)
Supplement: Supplementary file 2 — Supplementary Material [file 44172_2024_297_MOESM2_ESM.pdf]

Supplementary information

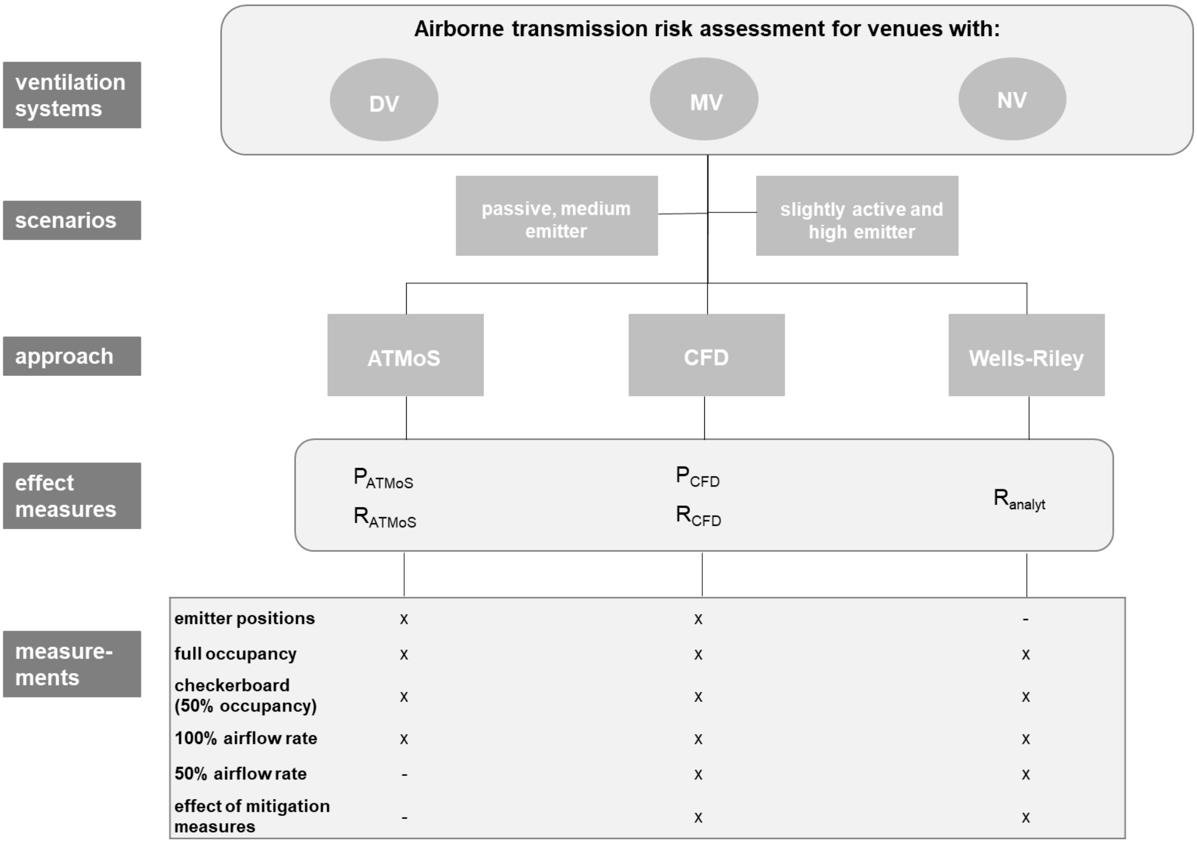

Figure S1: Flow chart of the study design

Venues with three different ventilation systems, namely displacement ventilation (DV), mixing ventilation (MV) and natural ventilation (NV) were assessed for their experimental (P_{ATMoS}, R_{ATMoS}), numerical (P_{CFD}, R_{CFD}) and analytical (P_{analyt}) individual and global risk of airborne transmission. Two emission profiles were considered for analyses: a sedentary, passive emitter with an average viral load and a slightly active emitter with a high viral load at the 90th percentile (high emitter). The effect of varying boundary conditions on the risk of infection was investigated, e.g. emitter position, occupancy rate, airflow rate, mitigation measures.

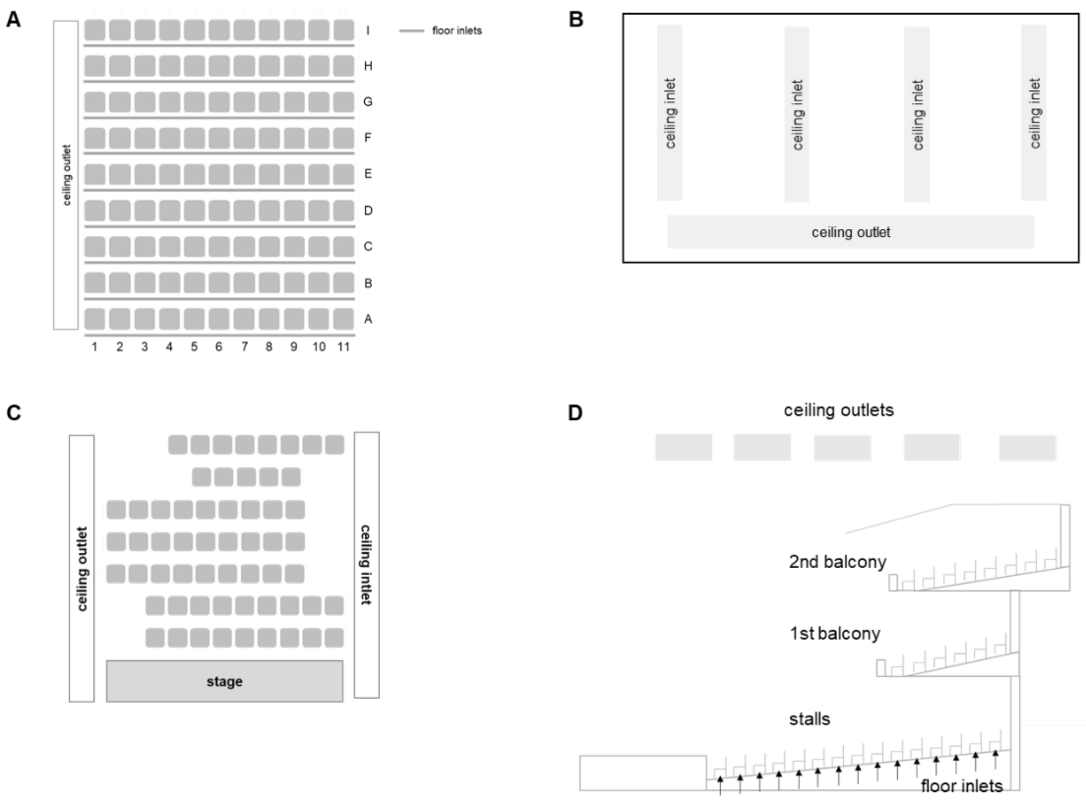

Figure S2: Positions of air inlets and outlets of the venues DVV (A), MVV (B), MVV2 (C) and HVV (D)

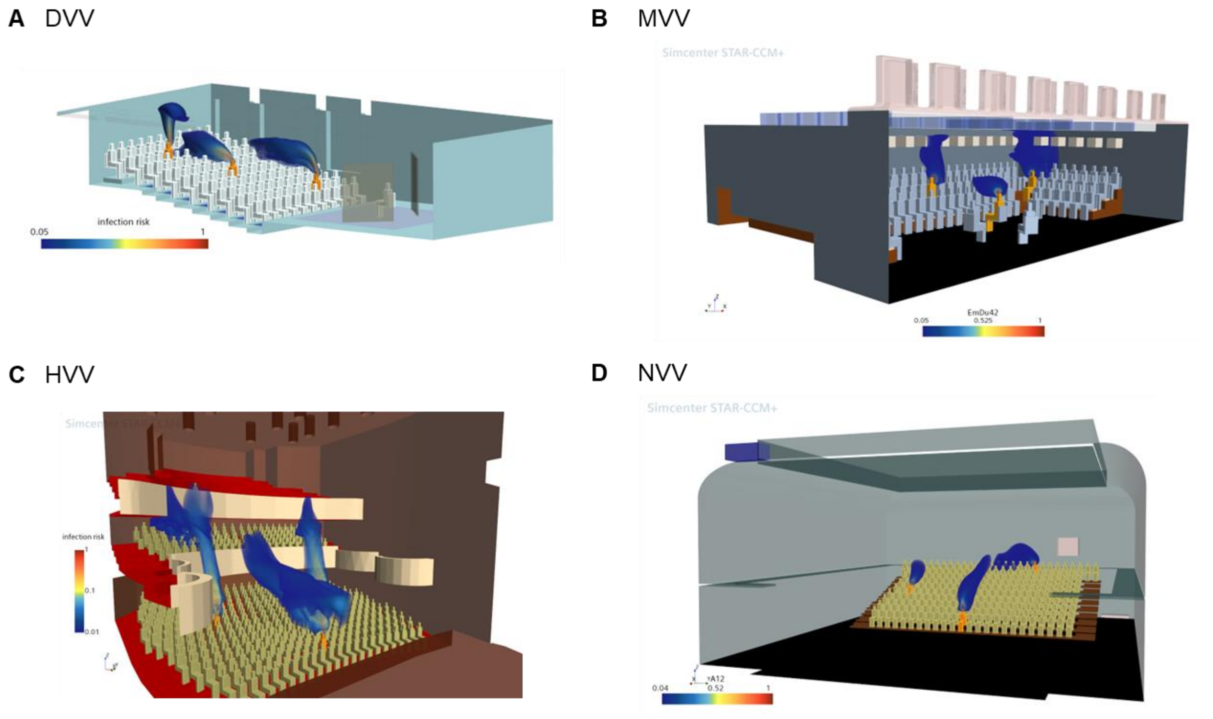

Figure S3: Numerical visualisation of the infection risk or relative contamination for DVV, MVV, HVV and NVV

Visualisation of the venue-specific infection risk for (A) DVV with emitter positions B9, E6 and H5, (B) MVV with emitter positions E16, H28 and I16, (C) HVV with emitter positions R8S21, R8S7, R2S15 and 1RR2S21 and (D) NVV with emitter positions A14, E5 and J20. Fig S3A, Fig. S3B and Fig. 3D were previously published in Schulz & Hehnen et al. (2024)[1].

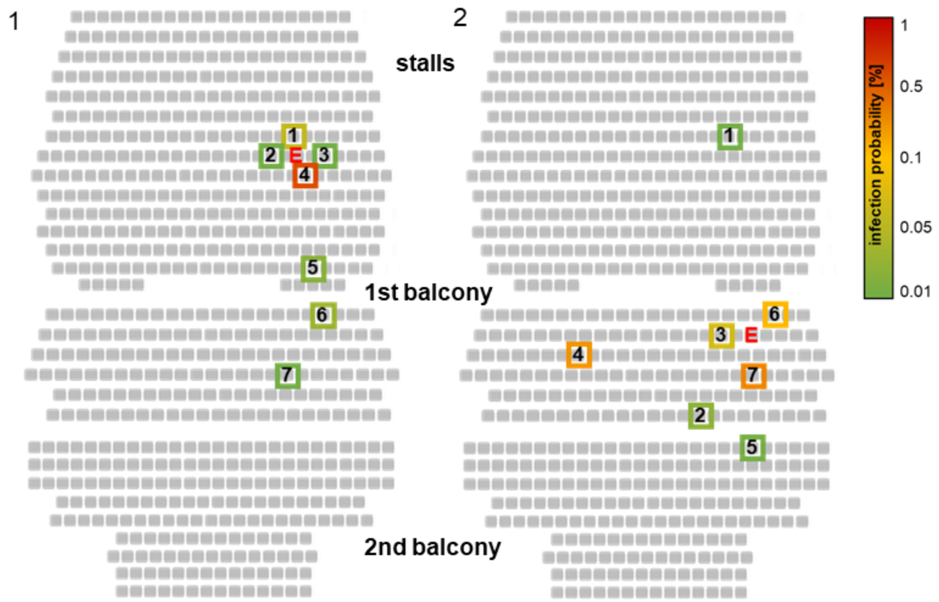

	absorber-specific P_{ATMoS}							R_{ATMoS}
	1	2	3	4	5	6	7	
stalls	0.06	0.00	0.00	0.66	0.02	0.03	0.00	0.52
1st balcony	0.00	0.02	0.07	0.26	0.00	0.13	0.37	0.58

Figure S4: Distribution of the experimentally derived individual and global risk of infection for the venue HVV with displacement ventilation in the stalls and unventilated balconies

The experimentally derived absorber-specific individual risk of infection P_{ATMoS} is shown for the emitter position R8S21 in the stalls (1) and for 1RR2S21 in the first rank (2), where "E" and coloured boxes indicate the positions of the emitter and the seven absorbers. The boxes of the absorbers are coloured according to their measured concentration with shades of green, yellow and red, representing low, medium and high measured values. P_{ATMoS} was calculated for each absorber using the absorbed NaCl mass and a quanta emission rate of 18.6 quanta h^{-1}. The values for each absorber are summarised in the table. The absorber-specific P_{ATMoS} values were used for the calculation of R_{ATMoS}.

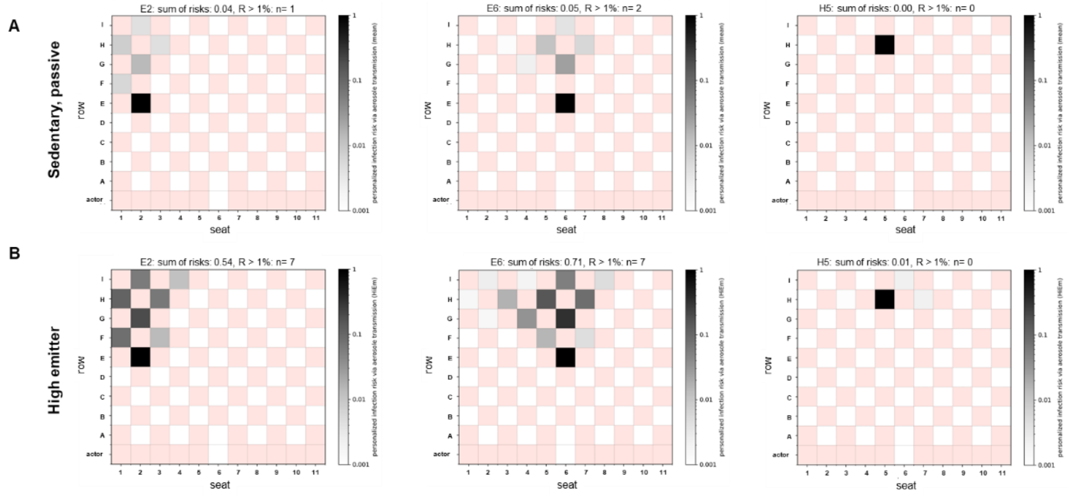

Figure S5: Influence of the checkerboard pattern seating arrangement on individual (P_{CFD}) and global risk of infection (R_{CFD}) at DVV

Infection risk plots for the emitter position E2, E6 and H5 for the silent passive emitter (A) and the high emitter (90th percentile; B) are shown with full air flow rate. The individual risk of infection is plotted for each position, except for the red positions as these do not represent seats in the audience. The sum of risk for each venue and emitter position as well as the number of spectators with R_{acc} > 1% are indicated above the plots and are summarised in the table.

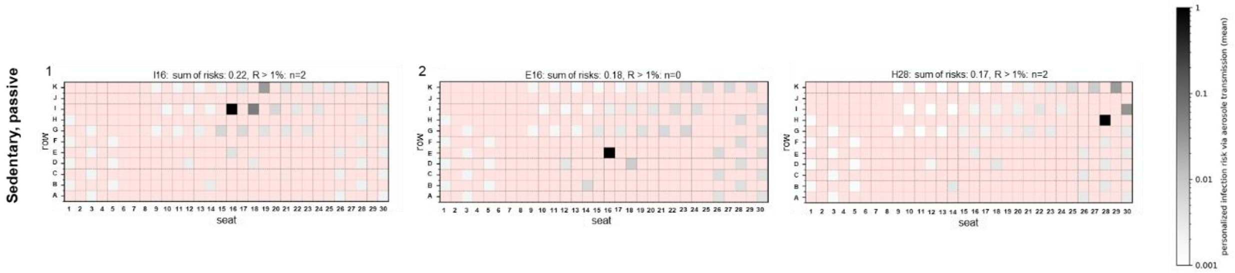

Figure S6: Distribution of the numerical individual (P_{CFD}) and global risk of infection (R_{CFD}) for MVV for a silent, passive emitter considering checkerboard seating arrangement

Infection risk plots for the emitter positions I16 (1), E16 (2) and H28 (3) are shown for a silent, passive emitter in a checkerboard arrangement of spectators. The individual risk of infection is plotted for each spectator, except for the red positions as these do not represent seats in the audience. The sum of risks for each venue and emitter position as well as the number of spectators with R_{acc} > 1% are indicated above the plots.

A E16

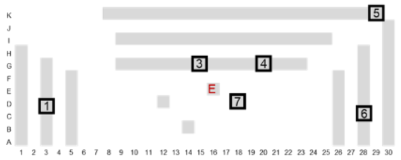

occupancy rate	absorber-specific P_{ATMoS}							R_{ATMoS}
	1	2	3	4	5	6	7	
100%	0.26	-	0.38	0.38	0.21	0.17	0.52	0.33
50%	0.10	-	0.30	0.52	0.15	0.23	0.49	0.16

B H28

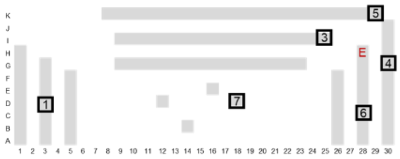

occupancy rate	absorber-specific P_{ATMoS}							R_{ATMoS}
	1	2	3	4	5	6	7	
100%	0.10	-	0.19	0.49	0.42	0.25	0.12	0.27
50%	0.04	-	0.04	0.49	0.60	0.33	0.13	0.14

C I16

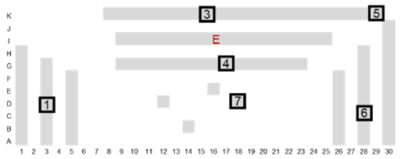

occupancy rate	absorber-specific P_{ATMoS}							R_{ATMoS}
	1	2	3	4	5	6	7	
100%	0.15	-	0.22	0.35	0.16	0.19	0.28	0.23
50%	0.08	-	0.56	0.43	0.21	0.23	0.41	0.16

D

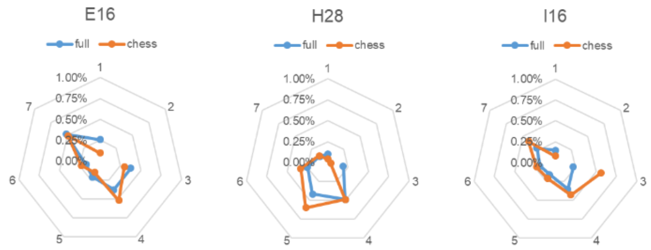

Figure S7: Influence of the checkerboard seating arrangement on experimentally derived individual (P_{ATMoS}) and global risk of infection (R_{ATMoS}) at MVV

The measurement positions for the experimentally derived individual infection risks P_{ATMoS} are shown as black boxes for the emitter ('E') positions E16 (A), H28 (B) and I16 (C) for the full occupancy and checkerboard seating arrangement for MVV. P_{ATMoS} was calculated for each absorber using the absorbed NaCl mass and a quanta emission rate of 18.6 quanta h^{-1}. The values for each absorber are summarised in the table. To obtain R_{ATMoS} the mean value of the seven absorber-specific P_{ATMoS} values of one measurement was calculated and multiplied by the total number of spectators. (D) P_{ATMoS} values for the seven absorbers are summarised in radar charts for each emitter position and occupancy configuration. The numbers 1 to 7 refer to the seven absorbers. The blue solid line showed the P_{ATMoS} values for full occupancy, the orange solid line represents the P_{ATMoS} values for the checkerboard pattern seating.

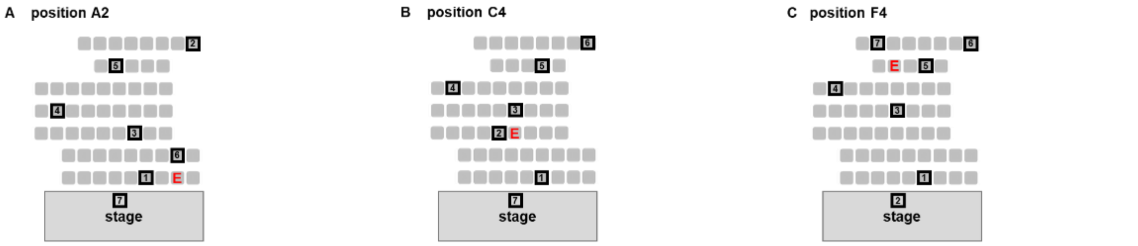

A (position A2)

occupancy rate	absorber-specific P_{ATMoS}							R_{ATMoS}
	1	2	3	4	5	6	7	
100%	0.23	-	0.40	0.20	0.20	0.44	0.12	0.16
50%	0.29	-	0.42	0.26	0.21	0.16	0.50	0.09

B (position C4)

occupancy rate	absorber-specific P_{ATMoS}							R_{ATMoS}
	1	2	3	4	5	6	7	
100%	0.07	0.47	0.10	0.06	0.10	0.10	0.09	0.09
50%	0.16	-	0.28	0.12	0.12	0.12	0.74	0.07

C (position F4)

occupancy rate	absorber-specific P_{ATMoS}							R_{ATMoS}
	1	2	3	4	5	6	7	
100%	0.11	-	0.13	0.16	0.21	0.28	0.16	0.10
50%	0.06	-	0.07	0.07	0.07	0.12	0.08	0.02

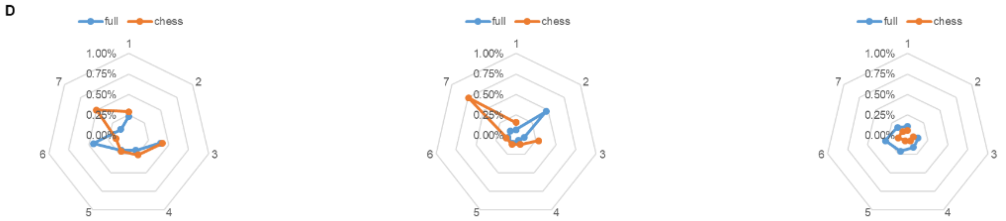

Figure S8: Influence of the checkerboard seating arrangement on experimentally derived individual (P_{ATMoS}) and global risk of infection (R_{ATMoS}) at MVV2

The measurement positions for the experimentally derived individual infection risks P_{ATMoS} are shown as black boxes for the emitter ('E') positions A2 (A), C4 (B) and F4 (C) for the full occupancy and checkerboard seating arrangement for MVV2. P_{ATMoS} was calculated for each absorber using the absorbed NaCl mass and a quanta emission rate of 18.6 quanta h^{-1}. The values for each absorber are summarised in the table. To obtain R_{ATMoS} the mean value of the seven absorber-specific P_{ATMoS} values of one measurement was calculated and multiplied by the total number of spectators. (D) P_{ATMoS} values for the seven absorbers are summarised in radar charts for each emitter position and occupancy configuration. The numbers 1 to 7 refer to the seven absorbers. The blue solid line showed the P_{ATMoS} values for full occupancy, the orange solid line represents the P_{ATMoS} values for the checkerboard pattern seating.

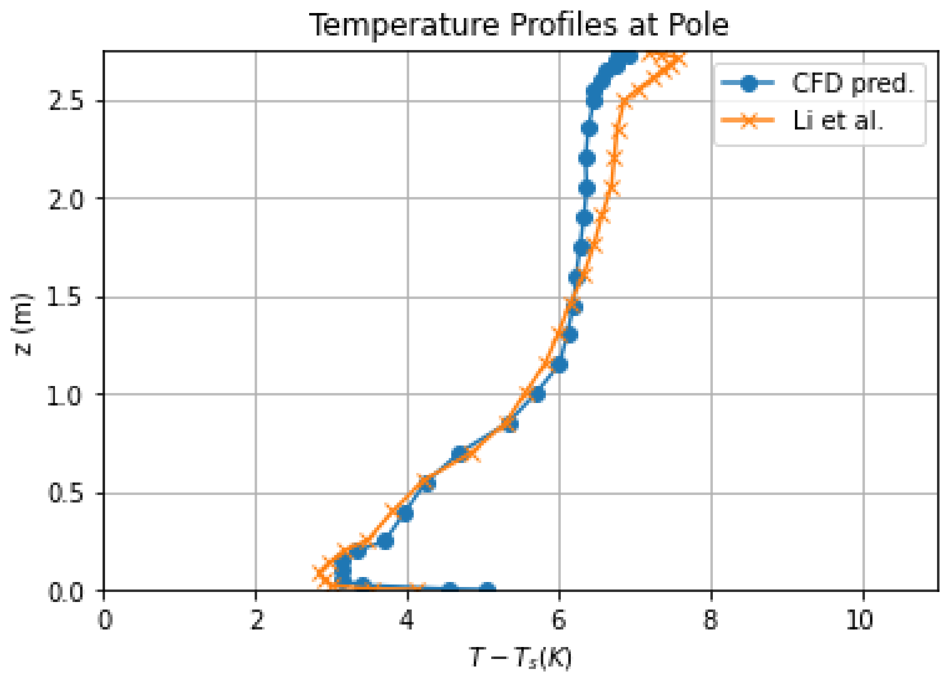

Figure S9: Comparison of the temperature profiles at the measurement pole along the height

The temperature differences along the pole are defined as in Li et al.[2,3]. The depicted case is given by condition B3 (300W heat load, n=3 volumes /hour). The temperature profile predicted by our CFD approach appears to be in good agreement except for the region just below the ceiling.

Table S1: Room characteristics of the investigated venues

Experimental measurement time and room characteristics of venues with displacement ventilation (DVV), with mixing ventilation (MVV, MVV2), with hybrid ventilation combining displacement and natural ventilation (HVV) and with natural ventilation (NVV) are listed. The introduction of the ventilation effectiveness E_z followed a similar approach of Sun & Zhai[4] and was adopted from ASHRAE Standard 62.1[5]. #Ventilation rate assumption for CFD analysis

Venue	Number of seats	Layout of auditorium	Volume of space [m³]	Room height [m]	Air flow rate [m³/h]	Ventilation effectiveness (E_z)	ATMoS measurement time [min]
**DVV**	99	ascending	530	3.9	4500	1.05	60
**HVV**	470	ascending	2300	10	15.000	1.5	57
**MVV**	103	ascending	400	3.3	6400	0.8	45
**MVV2**	60	ascending	650	5	3000	0.8	45
**NVV**	244	ascending	6000	10	1500#	0.5	27

Table S2: Parameter values for the computation of venue-specific risk tables

Parameter	Value	Unit
**Basic quanta emission rate silent, seating E_{P0}**	18.6	quanta h^{-1}
**breathing rate B_0 (susceptibles: silent, seating)**	0.486	m³/h
**relative quanta emission rate factor (r_E)**		
Silent	1	
singing/ shouting	30	
**relative breathing rate factor (r_E)**		
Silent	1	
singing/ shouting	1	
**quanta enhancement due to variants**	1	
Alpha	1.5	
Delta	2	
Omicron	3.3	
**face coverings**		
no (exhalation, inhalation)	1	
exhalation filtration efficiency	50	%
inhalation filtration efficiency	30	%
FFP2/N95 inh. /exh. filtration efficiency	80	%
**Contact time**		
short	1	h
medium	2	h
long	3	h
**Number of Infective people N_I**	1	

Table S3: Further NVV scenarios for maximum occupancy, event duration and acceptable risk
Evaluated cases for further approximations of maximum occupancy, event duration and acceptable risk per spectator in NVV are listed. The maximum values are distinguished between the sedentary, passive and the high emitting case and also between the three emitter positions. Furthermore, a maximum occupancy for a duration of 1.5h is calculated.

**Emission profile, quantity / emitter position**		**A14**	**E5**	**J20**
	quantity of interest			
**sedentary, passive**	R_{CFD}	2.63	2.02	2.57
-variable acceptable risk	$R_{\text{acc,reduced}} < \frac{1}{N}$			0.004
-variable number of spectators	N_{max}: $\frac{N_{\text{max}}}{N} R_{\text{CFD}} < 1$	92	120	94
-variable duration of event	t_{max}: $\sum_{i=1}^{N} P_{\text{CFD,n}}(\text{t}_{\text{max}}) < 1$	43 min	57 min	45 min
-variable number of spectators (1.5h duration)	R_{CFD}	2.00	1.53	1.95
	N_{max}: $\frac{N_{\text{max}}}{N} R_{\text{CFD}} < 1$	121	159	125
**high emitter**	R_{CFD}	36.79	28.46	36.02
-variable number of spectators	N_{max}: $\frac{N_{\text{max}}}{N} R_{\text{CFD}} < 1$	6	8	6
-variable duration of event	t_{max}: $\sum_{i=1}^{N} P_{\text{CFD,n}}(\text{t}_{\text{max}}) < 1$	3 min	4 min	3 min

Supplementary reference list

1. Schulz, I. et al. Experimental Device to Evaluate Aerosol Dispersion in Venues. *Applied Sciences* **14**, 5601 (2023).
2. Li, Y., Sandberg, M. & Fuchs, L. Effects of thermal radiation on airflow with displacement ventilation: an experimental investigation. *Energy Build* **19**, 263–274 (1993).
3. Li, Y., Sandberg, M. & Fuchs, L. Vertical Temperature Profiles in Rooms Ventilated by Displacement: Full-Scale Measurement and Nodal Modelling. *Indoor Air* **2**, 225–243 (1992).
4. Sun, C. & Zhai, Z. The efficacy of social distance and ventilation effectiveness in preventing COVID-19 transmission. Sustain Cities Soc 62, 102390 (2020).
5. ASHRAE Standards Committee. ANSI/ASHRAE Standard 62.1-2022 Ventilation and Acceptable Indoor Air Quality. 22 (2022).
